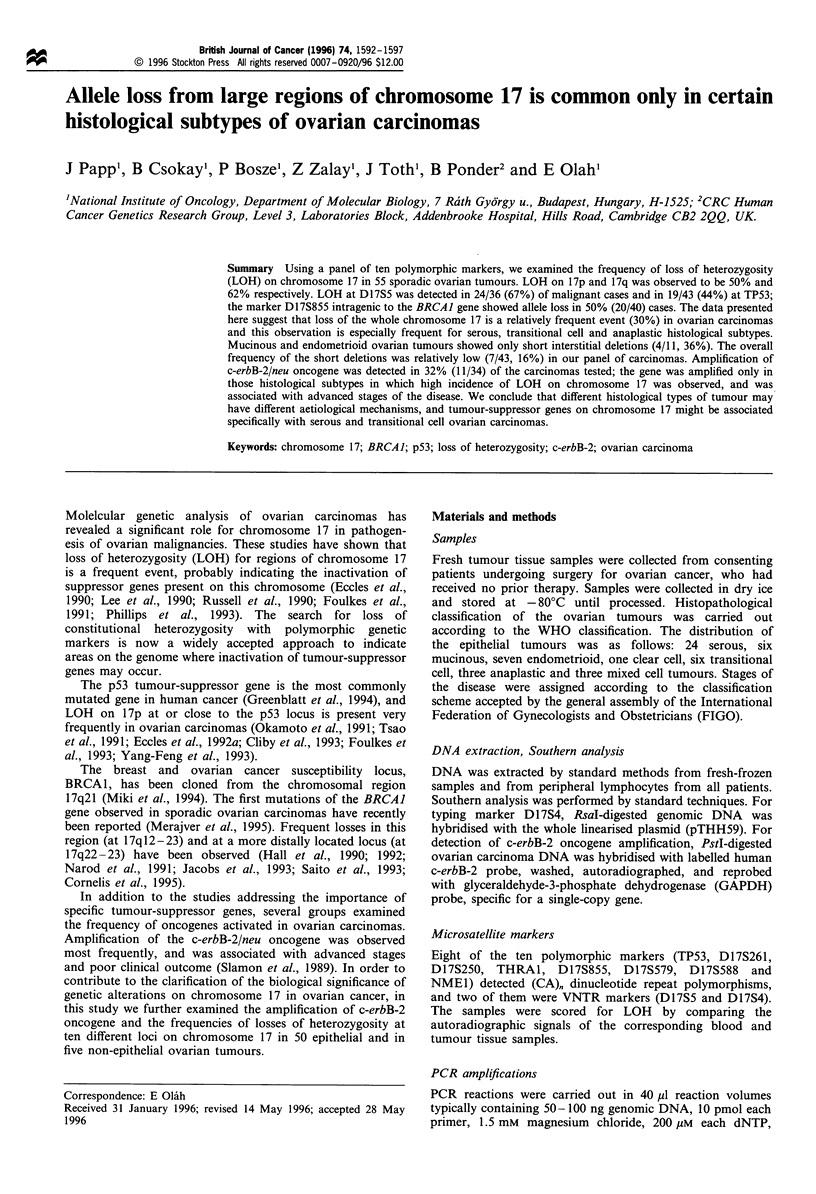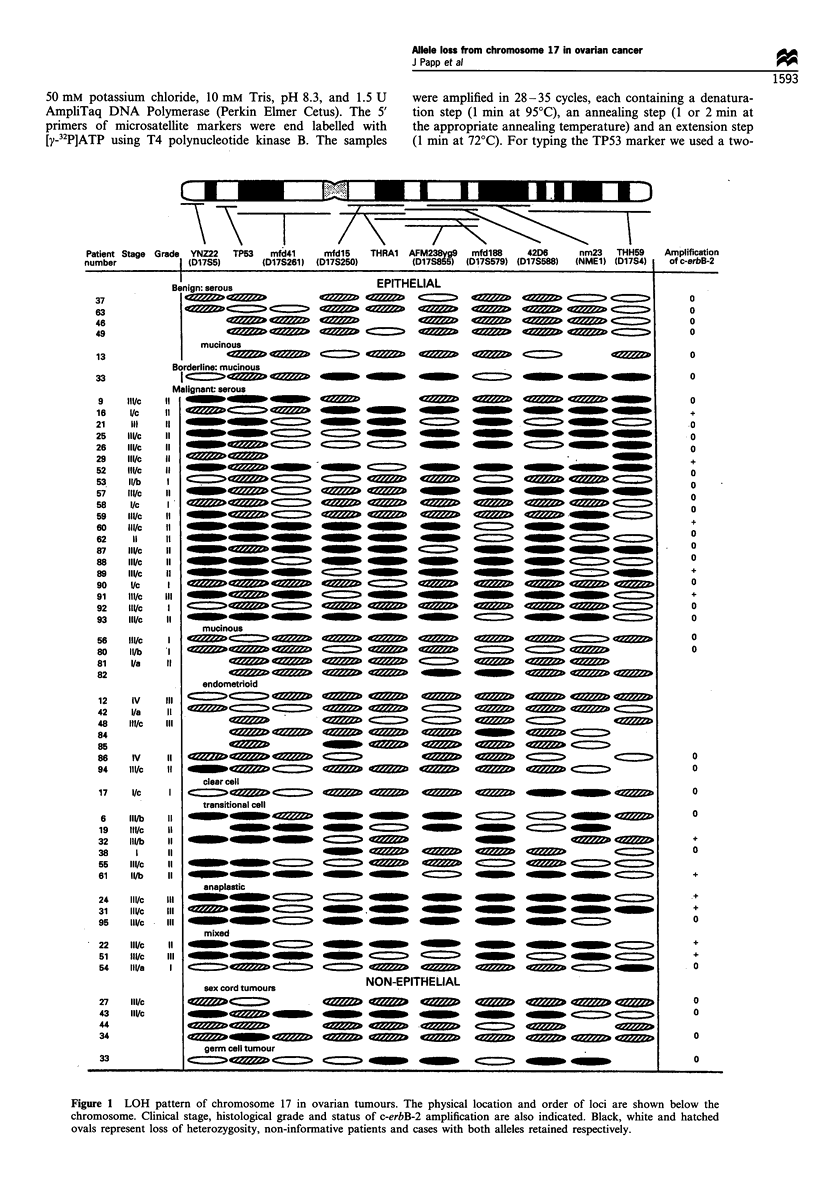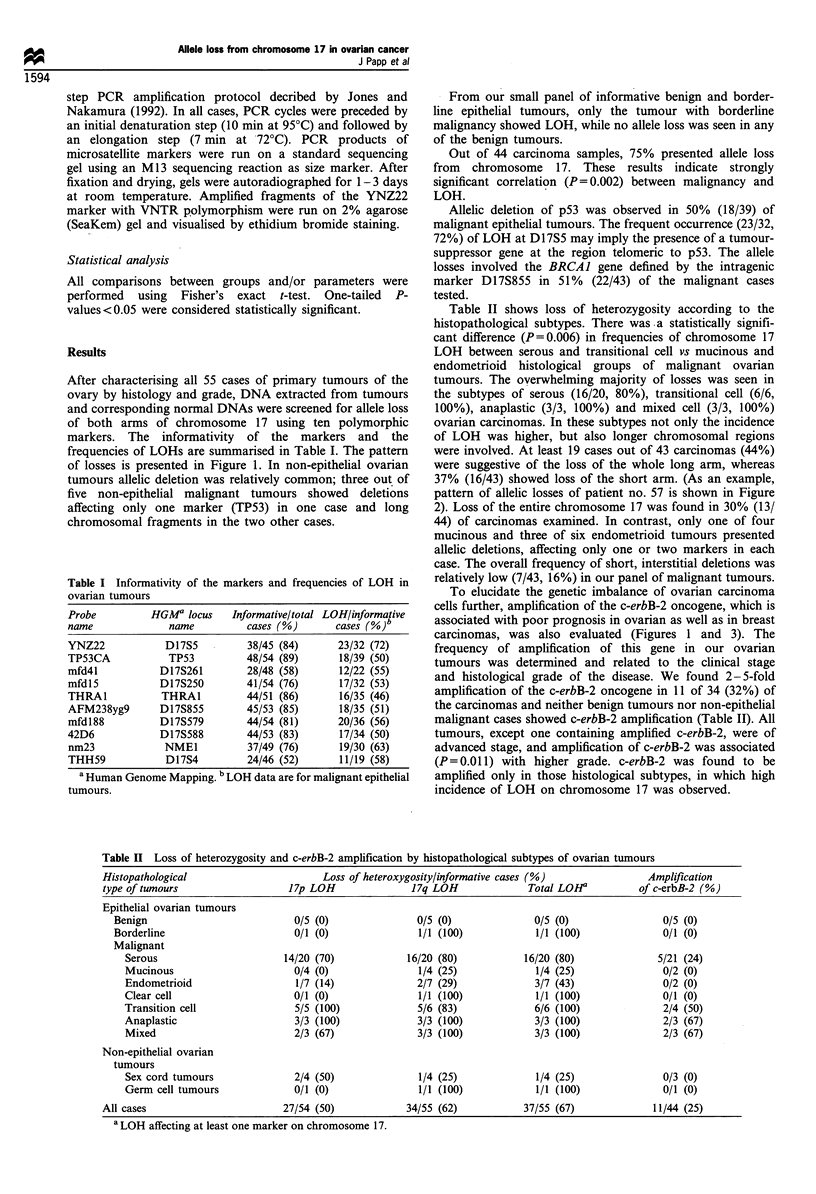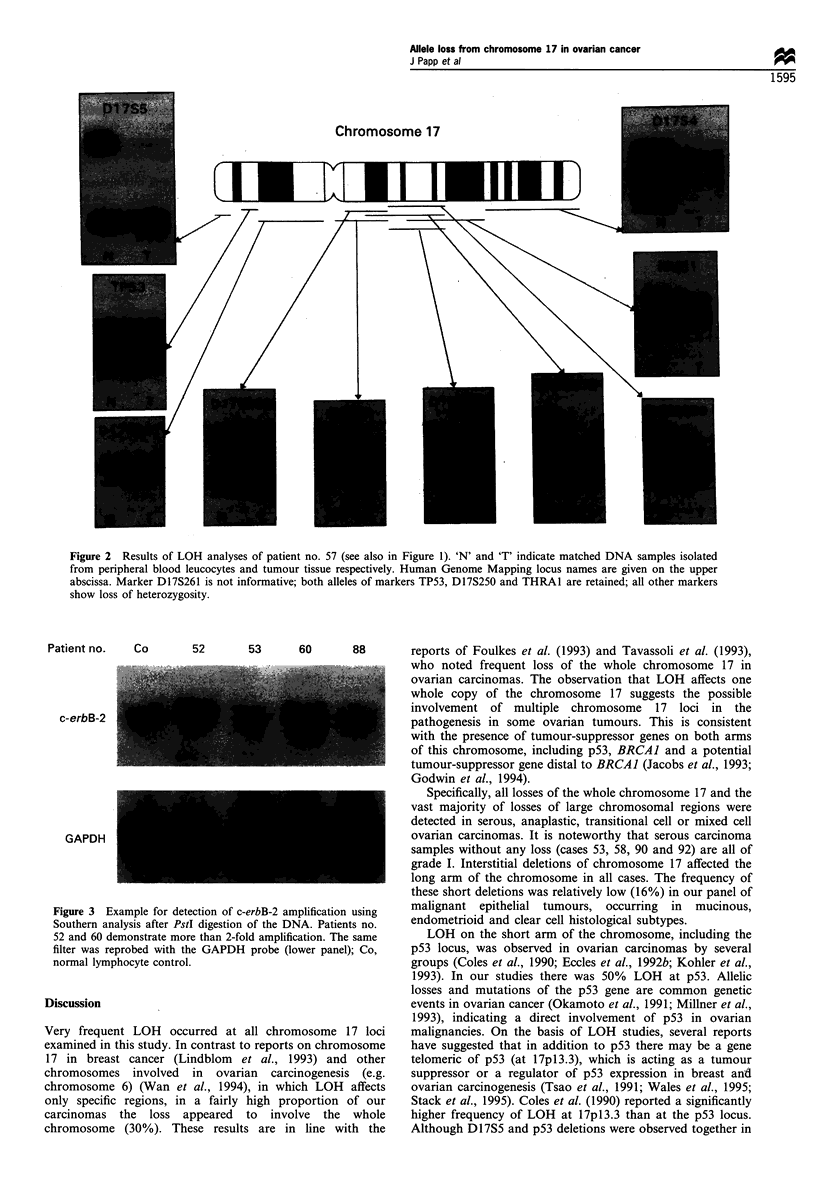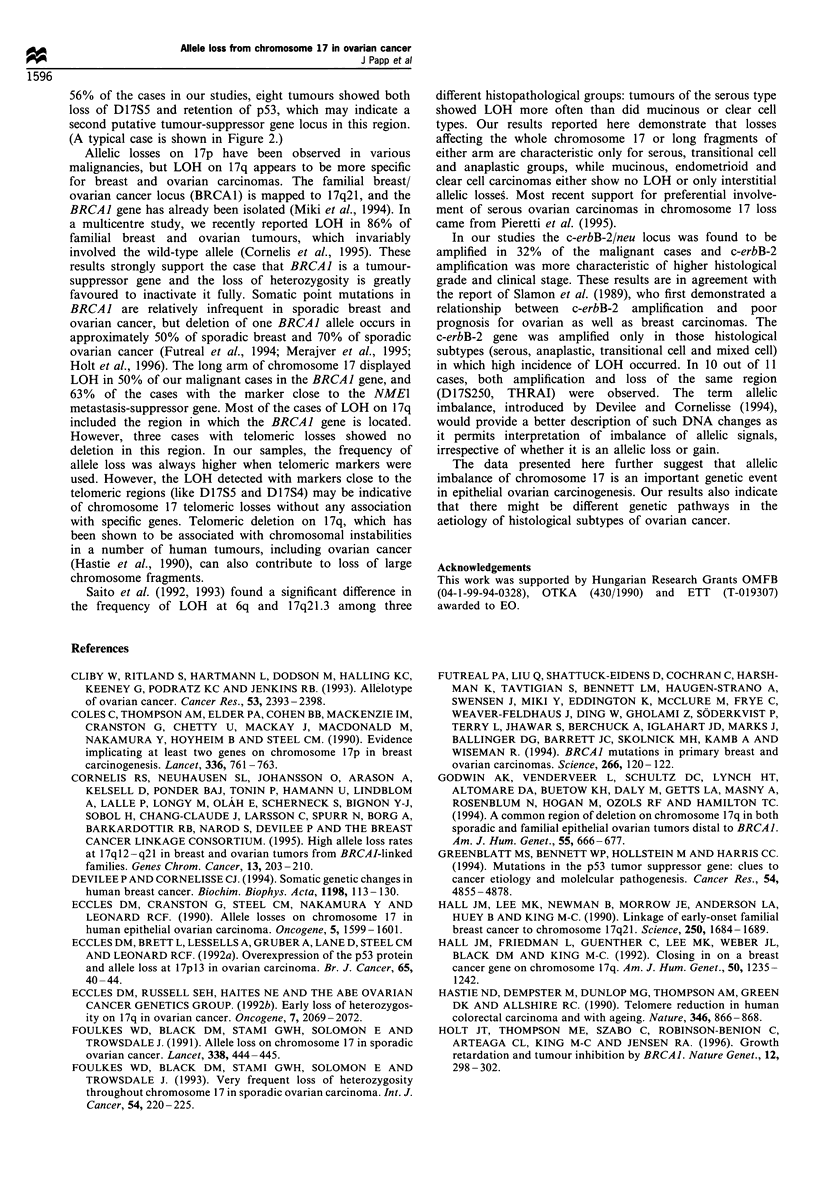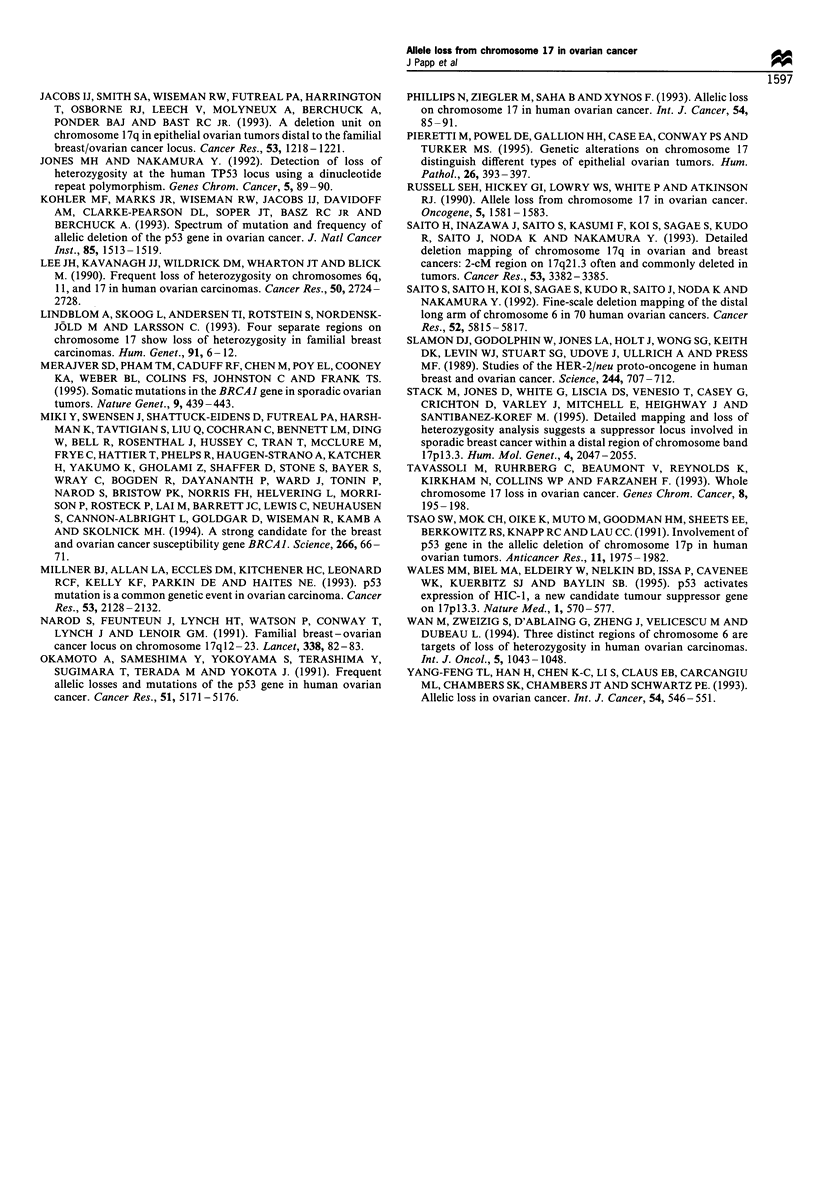# Allele loss from large regions of chromosome 17 is common only in certain histological subtypes of ovarian carcinomas.

**DOI:** 10.1038/bjc.1996.594

**Published:** 1996-11

**Authors:** J. Papp, B. Csokay, P. Bosze, Z. Zalay, J. Toth, B. Ponder, E. Olah

**Affiliations:** National Institute of Oncology, Department of Molecular Biology, Budapest, Hungary.

## Abstract

**Images:**


British Journal of Cancer (1996) 74, 1592-1597
© 1996 Stockton Press All rights reserved 0007-0920/96 $12.00

### Allele loss from large regions of chromosome 17 is common only in certain histological subtypes of ovarian carcinomas

J Papp[1], B Csokay[1], P Bosze[1], Z Zalay[1], J Toth[1], B Ponder[2] and E Olah[1]

[1]*National Institute of Oncology, Department of Molecular Biology, 7 Ráth György u., Budapest, Hungary, H-1525;* [2]*CRC Human Cancer Genetics Research Group, Level 3, Laboratories Block, Addenbrooke Hospital, Hills Road, Cambridge CB2 2QQ, UK.*

**Summary** Using a panel of ten polymorphic markers, we examined the frequency of loss of heterozygosity (LOH) on chromosome 17 in 55 sporadic ovarian tumours. LOH on 17p and 17q was observed to be 50% and 62% respectively. LOH at D17S5 was detected in 24/36 (67%) of malignant cases and in 19/43 (44%) at TP53; the marker D17S855 intragenic to the *BRCA1* gene showed allele loss in 50% (20/40) cases. The data presented here suggest that loss of the whole chromosome 17 is a relatively frequent event (30%) in ovarian carcinomas and this observation is especially frequent for serous, transitional cell and anaplastic histological subtypes. Mucinous and endometrioid ovarian tumours showed only short interstitial deletions (4/11, 36%). The overall frequency of the short deletions was relatively low (7/43, 16%) in our panel of carcinomas. Amplification of c-*erb*B-2/*neu* oncogene was detected in 32% (11/34) of the carcinomas tested; the gene was amplified only in those histological subtypes in which high incidence of LOH on chromosome 17 was observed, and was associated with advanced stages of the disease. We conclude that different histological types of tumour may have different aetiological mechanisms, and tumour-suppressor genes on chromosome 17 might be associated specifically with serous and transitional cell ovarian carcinomas.

**Keywords:** chromosome 17; *BRCA1*; p53; loss of heterozygosity; c-*erb*B-2; ovarian carcinoma

Molelcular genetic analysis of ovarian carcinomas has revealed a significant role for chromosome 17 in pathogenesis of ovarian malignancies. These studies have shown that loss of heterozygosity (LOH) for regions of chromosome 17 is a frequent event, probably indicating the inactivation of suppressor genes present on this chromosome (Eccles *et al.*, 1990; Lee *et al.*, 1990; Russell *et al.*, 1990; Foulkes *et al.*, 1991; Phillips *et al.*, 1993). The search for loss of constitutional heterozygosity with polymorphic genetic markers is now a widely accepted approach to indicate areas on the genome where inactivation of tumour-suppressor genes may occur.

The p53 tumour-suppressor gene is the most commonly mutated gene in human cancer (Greenblatt *et al.*, 1994), and LOH on 17p at or close to the p53 locus is present very frequently in ovarian carcinomas (Okamoto *et al.*, 1991; Tsao *et al.*, 1991; Eccles *et al.*, 1992*a*; Cliby *et al.*, 1993; Foulkes *et al.*, 1993; Yang-Feng *et al.*, 1993).

The breast and ovarian cancer susceptibility locus, BRCA1, has been cloned from the chromosomal region 17q21 (Miki *et al.*, 1994). The first mutations of the *BRCA1* gene observed in sporadic ovarian carcinomas have recently been reported (Merajver *et al.*, 1995). Frequent losses in this region (at 17q12–23) and at a more distally located locus (at 17q22–23) have been observed (Hall *et al.*, 1990; 1992; Narod *et al.*, 1991; Jacobs *et al.*, 1993; Saito *et al.*, 1993; Cornelis *et al.*, 1995).

In addition to the studies addressing the importance of specific tumour-suppressor genes, several groups examined the frequency of oncogenes activated in ovarian carcinomas. Amplification of the c-*erb*B-2/*neu* oncogene was observed most frequently, and was associated with advanced stages and poor clinical outcome (Slamon *et al.*, 1989). In order to contribute to the clarification of the biological significance of genetic alterations on chromosome 17 in ovarian cancer, in this study we further examined the amplification of c-*erb*B-2 oncogene and the frequencies of losses of heterozygosity at ten different loci on chromosome 17 in 50 epithelial and in five non-epithelial ovarian tumours.

Correspondence: E Oláh
Received 31 January 1996; revised 14 May 1996; accepted 28 May 1996

### Materials and methods

### Samples

Fresh tumour tissue samples were collected from consenting patients undergoing surgery for ovarian cancer, who had received no prior therapy. Samples were collected in dry ice and stored at −80°C until processed. Histopathological classification of the ovarian tumours was carried out according to the WHO classification. The distribution of the epithelial tumours was as follows: 24 serous, six mucinous, seven endometrioid, one clear cell, six transitional cell, three anaplastic and three mixed cell tumours. Stages of the disease were assigned according to the classification scheme accepted by the general assembly of the International Federation of Gynecologists and Obstetricians (FIGO).

### DNA extraction, Southern analysis

DNA was extracted by standard methods from fresh-frozen samples and from peripheral lymphocytes from all patients. Southern analysis was performed by standard techniques. For typing marker D17S4, *Rsa*I-digested genomic DNA was hybridised with the whole linearised plasmid (pTHH59). For detection of c-*erb*B-2 oncogene amplification, *Pst*I-digested ovarian carcinoma DNA was hybridised with labelled human c-*erb*B-2 probe, washed, autoradiographed, and reprobed with glyceraldehyde-3-phosphate dehydrogenase (GAPDH) probe, specific for a single-copy gene.

### Microsatellite markers

Eight of the ten polymorphic markers (TP53, D17S261, D17S250, THRA1, D17S855, D17S579, D17S588 and NME1) detected $(CA)_n$ dinucleotide repeat polymorphisms, and two of them were VNTR markers (D17S5 and D17S4). The samples were scored for LOH by comparing the autoradiographic signals of the corresponding blood and tumour tissue samples.

### PCR amplifications

PCR reactions were carried out in 40 $\mu$l reaction volumes typically containing 50–100 ng genomic DNA, 10 pmol each primer, 1.5 mM magnesium chloride, 200 $\mu$M each dNTP,

50 mM potassium chloride, 10 mM Tris, pH 8.3, and 1.5 U AmpliTaq DNA Polymerase (Perkin Elmer Cetus). The 5′ primers of microsatellite markers were end labelled with [$\gamma$-$^{32}$P]ATP using T4 polynucleotide kinase B. The samples were amplified in 28–35 cycles, each containing a denaturation step (1 min at 95°C), an annealing step (1 or 2 min at the appropriate annealing temperature) and an extension step (1 min at 72°C). For typing the TP53 marker we used a two-

**Figure 1** LOH pattern of chromosome 17 in ovarian tumours. The physical location and order of loci are shown below the chromosome. Clinical stage, histological grade and status of c-*erb*B-2 amplification are also indicated. Black, white and hatched ovals represent loss of heterozygosity, non-informative patients and cases with both alleles retained respectively.

step PCR amplification protocol decribed by Jones and Nakamura (1992). In all cases, PCR cycles were preceded by an initial denaturation step (10 min at 95°C) and followed by an elongation step (7 min at 72°C). PCR products of microsatellite markers were run on a standard sequencing gel using an M13 sequencing reaction as size marker. After fixation and drying, gels were autoradiographed for 1–3 days at room temperature. Amplified fragments of the YNZ22 marker with VNTR polymorphism were run on 2% agarose (SeaKem) gel and visualised by ethidium bromide staining.

*Statistical analysis*

All comparisons between groups and/or parameters were performed using Fisher's exact *t*-test. One-tailed *P*-values < 0.05 were considered statistically significant.

### Results

After characterising all 55 cases of primary tumours of the ovary by histology and grade, DNA extracted from tumours and corresponding normal DNAs were screened for allele loss of both arms of chromosome 17 using ten polymorphic markers. The informativity of the markers and the frequencies of LOHs are summarised in Table I. The pattern of losses is presented in Figure 1. In non-epithelial ovarian tumours allelic deletion was relatively common; three out of five non-epithelial malignant tumours showed deletions affecting only one marker (TP53) in one case and long chromosomal fragments in the two other cases.

**Table I** Informativity of the markers and frequencies of LOH in ovarian tumours

| *Probe name* | *HGM[a] locus name* | *Informative/total cases (%)* | *LOH/informative cases (%)[b]* |
|---|---|---|---|
| YNZ22 | D17S5 | 38/45 (84) | 23/32 (72) |
| TP53CA | TP53 | 48/54 (89) | 18/39 (50) |
| mfd41 | D17S261 | 28/48 (58) | 12/22 (55) |
| mfd15 | D17S250 | 41/54 (76) | 17/32 (53) |
| THRA1 | THRA1 | 44/51 (86) | 16/35 (46) |
| AFM238yg9 | D17S855 | 45/53 (85) | 18/35 (51) |
| mfd188 | D17S579 | 44/54 (81) | 20/36 (56) |
| 42D6 | D17S588 | 44/53 (83) | 17/34 (50) |
| nm23 | NME1 | 37/49 (76) | 19/30 (63) |
| THH59 | D17S4 | 24/46 (52) | 11/19 (58) |

[a] Human Genome Mapping. [b] LOH data are for malignant epithelial tumours.

From our small panel of informative benign and borderline epithelial tumours, only the tumour with borderline malignancy showed LOH, while no allele loss was seen in any of the benign tumours.

Out of 44 carcinoma samples, 75% presented allele loss from chromosome 17. These results indicate strongly significant correlation ($P = 0.002$) between malignancy and LOH.

Allelic deletion of p53 was observed in 50% (18/39) of malignant epithelial tumours. The frequent occurrence (23/32, 72%) of LOH at D17S5 may imply the presence of a tumour-suppressor gene at the region telomeric to p53. The allele losses involved the *BRCA1* gene defined by the intragenic marker D17S855 in 51% (22/43) of the malignant cases tested.

Table II shows loss of heterozygosity according to the histopathological subtypes. There was a statistically significant difference ($P = 0.006$) in frequencies of chromosome 17 LOH between serous and transitional cell *vs* mucinous and endometrioid histological groups of malignant ovarian tumours. The overwhelming majority of losses was seen in the subtypes of serous (16/20, 80%), transitional cell (6/6, 100%), anaplastic (3/3, 100%) and mixed cell (3/3, 100%) ovarian carcinomas. In these subtypes not only the incidence of LOH was higher, but also longer chromosomal regions were involved. At least 19 cases out of 43 carcinomas (44%) were suggestive of the loss of the whole long arm, whereas 37% (16/43) showed loss of the short arm. (As an example, pattern of allelic losses of patient no. 57 is shown in Figure 2). Loss of the entire chromosome 17 was found in 30% (13/44) of carcinomas examined. In contrast, only one of four mucinous and three of six endometrioid tumours presented allelic deletions, affecting only one or two markers in each case. The overall frequency of short, interstitial deletions was relatively low (7/43, 16%) in our panel of malignant tumours.

To elucidate the genetic imbalance of ovarian carcinoma cells further, amplification of the c-*erb*B-2 oncogene, which is associated with poor prognosis in ovarian as well as in breast carcinomas, was also evaluated (Figures 1 and 3). The frequency of amplification of this gene in our ovarian tumours was determined and related to the clinical stage and histological grade of the disease. We found 2–5-fold amplification of the c-*erb*B-2 oncogene in 11 of 34 (32%) of the carcinomas and neither benign tumours nor non-epithelial malignant cases showed c-*erb*B-2 amplification (Table II). All tumours, except one containing amplified c-*erb*B-2, were of advanced stage, and amplification of c-*erb*B-2 was associated ($P = 0.011$) with higher grade. c-*erb*B-2 was found to be amplified only in those histological subtypes, in which high incidence of LOH on chromosome 17 was observed.

**Table II** Loss of heterozygosity and c-*erb*B-2 amplification by histopathological subtypes of ovarian tumours

| *Histopathological type of tumours* | *Loss of heteroxygosity/informative cases (%)* 17p LOH | 17q LOH | Total LOH[a] | *Amplification of c-erbB-2 (%)* |
|---|---|---|---|---|
| Epithelial ovarian tumours | | | | |
| Benign | 0/5 (0) | 0/5 (0) | 0/5 (0) | 0/5 (0) |
| Borderline | 0/1 (0) | 1/1 (100) | 1/1 (100) | 0/1 (0) |
| Malignant | | | | |
| Serous | 14/20 (70) | 16/20 (80) | 16/20 (80) | 5/21 (24) |
| Mucinous | 0/4 (0) | 1/4 (25) | 1/4 (25) | 0/2 (0) |
| Endometrioid | 1/7 (14) | 2/7 (29) | 3/7 (43) | 0/2 (0) |
| Clear cell | 0/1 (0) | 1/1 (100) | 1/1 (100) | 0/1 (0) |
| Transition cell | 5/5 (100) | 5/6 (83) | 6/6 (100) | 2/4 (50) |
| Anaplastic | 3/3 (100) | 3/3 (100) | 3/3 (100) | 2/3 (67) |
| Mixed | 2/3 (67) | 3/3 (100) | 3/3 (100) | 2/3 (67) |
| Non-epithelial ovarian tumours | | | | |
| Sex cord tumours | 2/4 (50) | 1/4 (25) | 1/4 (25) | 0/3 (0) |
| Germ cell tumours | 0/1 (0) | 1/1 (100) | 1/1 (100) | 0/1 (0) |
| All cases | 27/54 (50) | 34/55 (62) | 37/55 (67) | 11/44 (25) |

[a] LOH affecting at least one marker on chromosome 17.

**Figure 2** Results of LOH analyses of patient no. 57 (see also in Figure 1). 'N' and 'T' indicate matched DNA samples isolated from peripheral blood leucocytes and tumour tissue respectively. Human Genome Mapping locus names are given on the upper abscissa. Marker D17S261 is not informative; both alleles of markers TP53, D17S250 and THRA1 are retained; all other markers show loss of heterozygosity.

**Figure 3** Example for detection of c-*erb*B-2 amplification using Southern analysis after *Pst*I digestion of the DNA. Patients no. 52 and 60 demonstrate more than 2-fold amplification. The same filter was reprobed with the GAPDH probe (lower panel); Co, normal lymphocyte control.

### Discussion

Very frequent LOH occurred at all chromosome 17 loci examined in this study. In contrast to reports on chromosome 17 in breast cancer (Lindblom *et al.*, 1993) and other chromosomes involved in ovarian carcinogenesis (e.g. chromosome 6) (Wan *et al.*, 1994), in which LOH affects only specific regions, in a fairly high proportion of our carcinomas the loss appeared to involve the whole chromosome (30%). These results are in line with the reports of Foulkes *et al.* (1993) and Tavassoli *et al.* (1993), who noted frequent loss of the whole chromosome 17 in ovarian carcinomas. The observation that LOH affects one whole copy of the chromosome 17 suggests the possible involvement of multiple chromosome 17 loci in the pathogenesis in some ovarian tumours. This is consistent with the presence of tumour-suppressor genes on both arms of this chromosome, including p53, *BRCA1* and a potential tumour-suppressor gene distal to *BRCA1* (Jacobs *et al.*, 1993; Godwin *et al.*, 1994).

Specifically, all losses of the whole chromosome 17 and the vast majority of losses of large chromosomal regions were detected in serous, anaplastic, transitional cell or mixed cell ovarian carcinomas. It is noteworthy that serous carcinoma samples without any loss (cases 53, 58, 90 and 92) are all of grade I. Interstitial deletions of chromosome 17 affected the long arm of the chromosome in all cases. The frequency of these short deletions was relatively low (16%) in our panel of malignant epithelial tumours, occurring in mucinous, endometrioid and clear cell histological subtypes.

LOH on the short arm of the chromosome, including the p53 locus, was observed in ovarian carcinomas by several groups (Coles *et al.*, 1990; Eccles *et al.*, 1992*b*; Kohler *et al.*, 1993). In our studies there was 50% LOH at p53. Allelic losses and mutations of the p53 gene are common genetic events in ovarian cancer (Okamoto *et al.*, 1991; Millner *et al.*, 1993), indicating a direct involvement of p53 in ovarian malignancies. On the basis of LOH studies, several reports have suggested that in addition to p53 there may be a gene telomeric of p53 (at 17p13.3), which is acting as a tumour suppressor or a regulator of p53 expression in breast and ovarian carcinogenesis (Tsao *et al.*, 1991; Wales *et al.*, 1995; Stack *et al.*, 1995). Coles *et al.* (1990) reported a significantly higher frequency of LOH at 17p13.3 than at the p53 locus. Although D17S5 and p53 deletions were observed together in

56% of the cases in our studies, eight tumours showed both loss of D17S5 and retention of p53, which may indicate a second putative tumour-suppressor gene locus in this region. (A typical case is shown in Figure 2.)

Allelic losses on 17p have been observed in various malignancies, but LOH on 17q appears to be more specific for breast and ovarian carcinomas. The familial breast/ovarian cancer locus (BRCA1) is mapped to 17q21, and the *BRCA1* gene has already been isolated (Miki *et al.*, 1994). In a multicentre study, we recently reported LOH in 86% of familial breast and ovarian tumours, which invariably involved the wild-type allele (Cornelis *et al.*, 1995). These results strongly support the case that *BRCA1* is a tumour-suppressor gene and the loss of heterozygosity is greatly favoured to inactivate it fully. Somatic point mutations in *BRCA1* are relatively infrequent in sporadic breast and ovarian cancer, but deletion of one *BRCA1* allele occurs in approximately 50% of sporadic breast and 70% of sporadic ovarian cancer (Futreal *et al.*, 1994; Merajver *et al.*, 1995; Holt *et al.*, 1996). The long arm of chromosome 17 displayed LOH in 50% of our malignant cases in the *BRCA1* gene, and 63% of the cases with the marker close to the *NME*1 metastasis-suppressor gene. Most of the cases of LOH on 17q included the region in which the *BRCA1* gene is located. However, three cases with telomeric losses showed no deletion in this region. In our samples, the frequency of allele loss was always higher when telomeric markers were used. However, the LOH detected with markers close to the telomeric regions (like D17S5 and D17S4) may be indicative of chromosome 17 telomeric losses without any association with specific genes. Telomeric deletion on 17q, which has been shown to be associated with chromosomal instabilities in a number of human tumours, including ovarian cancer (Hastie *et al.*, 1990), can also contribute to loss of large chromosome fragments.

Saito *et al.* (1992, 1993) found a significant difference in the frequency of LOH at 6q and 17q21.3 among three different histopathological groups: tumours of the serous type showed LOH more often than did mucinous or clear cell types. Our results reported here demonstrate that losses affecting the whole chromosome 17 or long fragments of either arm are characteristic only for serous, transitional cell and anaplastic groups, while mucinous, endometrioid and clear cell carcinomas either show no LOH or only interstitial allelic losses. Most recent support for preferential involvement of serous ovarian carcinomas in chromosome 17 loss came from Pieretti *et al.* (1995).

In our studies the c-*erb*B-2/*neu* locus was found to be amplified in 32% of the malignant cases and c-*erb*B-2 amplification was more characteristic of higher histological grade and clinical stage. These results are in agreement with the report of Slamon *et al.* (1989), who first demonstrated a relationship between c-*erb*B-2 amplification and poor prognosis for ovarian as well as breast carcinomas. The c-*erb*B-2 gene was amplified only in those histological subtypes (serous, anaplastic, transitional cell and mixed cell) in which high incidence of LOH occurred. In 10 out of 11 cases, both amplification and loss of the same region (D17S250, THRAI) were observed. The term allelic imbalance, introduced by Devilee and Cornelisse (1994), would provide a better description of such DNA changes as it permits interpretation of imbalance of allelic signals, irrespective of whether it is an allelic loss or gain.

The data presented here further suggest that allelic imbalance of chromosome 17 is an important genetic event in epithelial ovarian carcinogenesis. Our results also indicate that there might be different genetic pathways in the aetiology of histological subtypes of ovarian cancer.

**Acknowledgements**
This work was supported by Hungarian Research Grants OMFB (04-1-99-94-0328), OTKA (430/1990) and ETT (T-019307) awarded to EO.

### References

CLIBY W, RITLAND S, HARTMANN L, DODSON M, HALLING KC, KEENEY G, PODRATZ KC AND JENKINS RB. (1993). Allelotype of ovarian cancer. *Cancer Res.*, **53**, 2393–2398.

COLES C, THOMPSON AM, ELDER PA, COHEN BB, MACKENZIE IM, CRANSTON G, CHETTY U, MACKAY J, MACDONALD M, NAKAMURA Y, HOYHEIM B AND STEEL CM. (1990). Evidence implicating at least two genes on chromosome 17p in breast carcinogenesis. *Lancet*, **336**, 761–763.

CORNELIS RS, NEUHAUSEN SL, JOHANSSON O, ARASON A, KELSELL D, PONDER BAJ, TONIN P, HAMANN U, LINDBLOM A, LALLE P, LONGY M, OLÁH E, SCHERNECK S, BIGNON Y-J, SOBOL H, CHANG-CLAUDE J, LARSSON C, SPURR N, BORG A, BARKARDOTTIR RB, NAROD S, DEVILEE P AND THE BREAST CANCER LINKAGE CONSORTIUM. (1995). High allele loss rates at 17q12–q21 in breast and ovarian tumors from *BRCA1*-linked families. *Genes Chrom. Cancer*, **13**, 203–210.

DEVILEE P AND CORNELISSE CJ. (1994). Somatic genetic changes in human breast cancer. *Biochim. Biophys. Acta*, **1198**, 113–130.

ECCLES DM, CRANSTON G, STEEL CM, NAKAMURA Y AND LEONARD RCF. (1990). Allele losses on chromosome 17 in human epithelial ovarian carcinoma. *Oncogene*, **5**, 1599–1601.

ECCLES DM, BRETT L, LESSELLS A, GRUBER A, LANE D, STEEL CM AND LEONARD RCF. (1992*a*). Overexpression of the p53 protein and allele loss at 17p13 in ovarian carcinoma. *Br. J. Cancer*, **65**, 40–44.

ECCLES DM, RUSSELL SEH, HAITES NE AND THE ABE OVARIAN CANCER GENETICS GROUP. (1992*b*). Early loss of heterozygosity on 17q in ovarian cancer. *Oncogene*, **7**, 2069–2072.

FOULKES WD, BLACK DM, STAMI GWH, SOLOMON E AND TROWSDALE J. (1991). Allele loss on chromosome 17 in sporadic ovarian cancer. *Lancet*, **338**, 444–445.

FOULKES WD, BLACK DM, STAMI GWH, SOLOMON E AND TROWSDALE J. (1993). Very frequent loss of heterozygosity throughout chromosome 17 in sporadic ovarian carcinoma. *Int. J. Cancer*, **54**, 220–225.

FUTREAL PA, LIU Q, SHATTUCK-EIDENS D, COCHRAN C, HARSHMAN K, TAVTIGIAN S, BENNETT LM, HAUGEN-STRANO A, SWENSEN J, MIKI Y, EDDINGTON K, McCLURE M, FRYE C, WEAVER-FELDHAUS J, DING W, GHOLAMI Z, SÖDERKVIST P, TERRY L, JHAWAR S, BERCHUCK A, IGLAHART JD, MARKS J, BALLINGER DG, BARRETT JC, SKOLNICK MH, KAMB A AND WISEMAN R. (1994). *BRCA1* mutations in primary breast and ovarian carcinomas. *Science*, **266**, 120–122.

GODWIN AK, VENDERVEER L, SCHULTZ DC, LYNCH HT, ALTOMARE DA, BUETOW KH, DALY M, GETTS LA, MASNY A, ROSENBLUM N, HOGAN M, OZOLS RF AND HAMILTON TC. (1994). A common region of deletion on chromosome 17q in both sporadic and familial epithelial ovarian tumors distal to *BRCA1*. *Am. J. Hum. Genet.*, **55**, 666–677.

GREENBLATT MS, BENNETT WP, HOLLSTEIN M AND HARRIS CC. (1994). Mutations in the p53 tumor suppressor gene: clues to cancer etiology and molelcular pathogenesis. *Cancer Res.*, **54**, 4855–4878.

HALL JM, LEE MK, NEWMAN B, MORROW JE, ANDERSON LA, HUEY B AND KING M-C. (1990). Linkage of early-onset familial breast cancer to chromosome 17q21. *Science*, **250**, 1684–1689.

HALL JM, FRIEDMAN L, GUENTHER C, LEE MK, WEBER JL, BLACK DM AND KING M-C. (1992). Closing in on a breast cancer gene on chromosome 17q. *Am. J. Hum. Genet.*, **50**, 1235–1242.

HASTIE ND, DEMPSTER M, DUNLOP MG, THOMPSON AM, GREEN DK AND ALLSHIRE RC. (1990). Telomere reduction in human colorectal carcinoma and with ageing. *Nature*, **346**, 866–868.

HOLT JT, THOMPSON ME, SZABO C, ROBINSON-BENION C, ARTEAGA CL, KING M-C AND JENSEN RA. (1996). Growth retardation and tumour inhibition by *BRCA1*. *Nature Genet.*, **12**, 298–302.

JACOBS IJ, SMITH SA, WISEMAN RW, FUTREAL PA, HARRINGTON T, OSBORNE RJ, LEECH V, MOLYNEUX A, BERCHUCK A, PONDER BAJ AND BAST RC JR. (1993). A deletion unit on chromosome 17q in epithelial ovarian tumors distal to the familial breast/ovarian cancer locus. *Cancer Res.*, **53**, 1218–1221.

JONES MH AND NAKAMURA Y. (1992). Detection of loss of heterozygosity at the human TP53 locus using a dinucleotide repeat polymorphism. *Genes Chrom. Cancer*, **5**, 89–90.

KOHLER MF, MARKS JR, WISEMAN RW, JACOBS IJ, DAVIDOFF AM, CLARKE-PEARSON DL, SOPER JT, BASZ RC JR AND BERCHUCK A. (1993). Spectrum of mutation and frequency of allelic deletion of the p53 gene in ovarian cancer. *J. Natl Cancer Inst.*, **85**, 1513–1519.

LEE JH, KAVANAGH JJ, WILDRICK DM, WHARTON JT AND BLICK M. (1990). Frequent loss of heterozygosity on chromosomes 6q, 11, and 17 in human ovarian carcinomas. *Cancer Res.*, **50**, 2724–2728.

LINDBLOM A, SKOOG L, ANDERSEN TI, ROTSTEIN S, NORDENSKJÖLD M AND LARSSON C. (1993). Four separate regions on chromosome 17 show loss of heterozygosity in familial breast carcinomas. *Hum. Genet.*, **91**, 6–12.

MERAJVER SD, PHAM TM, CADUFF RF, CHEN M, POY EL, COONEY KA, WEBER BL, COLINS FS, JOHNSTON C AND FRANK TS. (1995). Somatic mutations in the *BRCA1* gene in sporadic ovarian tumors. *Nature Genet.*, **9**, 439–443.

MIKI Y, SWENSEN J, SHATTUCK-EIDENS D, FUTREAL PA, HARSHMAN K, TAVTIGIAN S, LIU Q, COCHRAN C, BENNETT LM, DING W, BELL R, ROSENTHAL J, HUSSEY C, TRAN T, MCCLURE M, FRYE C, HATTIER T, PHELPS R, HAUGEN-STRANO A, KATCHER H, YAKUMO K, GHOLAMI Z, SHAFFER D, STONE S, BAYER S, WRAY C, BOGDEN R, DAYANANTH P, WARD J, TONIN P, NAROD S, BRISTOW PK, NORRIS FH, HELVERING L, MORRISON P, ROSTECK P, LAI M, BARRETT JC, LEWIS C, NEUHAUSEN S, CANNON-ALBRIGHT L, GOLDGAR D, WISEMAN R, KAMB A AND SKOLNICK MH. (1994). A strong candidate for the breast and ovarian cancer susceptibility gene *BRCA1*. *Science*, **266**, 66–71.

MILLNER BJ, ALLAN LA, ECCLES DM, KITCHENER HC, LEONARD RCF, KELLY KF, PARKIN DE AND HAITES NE. (1993). p53 mutation is a common genetic event in ovarian carcinoma. *Cancer Res.*, **53**, 2128–2132.

NAROD S, FEUNTEUN J, LYNCH HT, WATSON P, CONWAY T, LYNCH J AND LENOIR GM. (1991). Familial breast–ovarian cancer locus on chromosome 17q12–23. *Lancet*, **338**, 82–83.

OKAMOTO A, SAMESHIMA Y, YOKOYAMA S, TERASHIMA Y, SUGIMARA T, TERADA M AND YOKOTA J. (1991). Frequent allelic losses and mutations of the p53 gene in human ovarian cancer. *Cancer Res.*, **51**, 5171–5176.

PHILLIPS N, ZIEGLER M, SAHA B AND XYNOS F. (1993). Allelic loss on chromosome 17 in human ovarian cancer. *Int. J. Cancer*, **54**, 85–91.

PIERETTI M, POWEL DE, GALLION HH, CASE EA, CONWAY PS AND TURKER MS. (1995). Genetic alterations on chromosome 17 distinguish different types of epithelial ovarian tumors. *Hum. Pathol.*, **26**, 393–397.

RUSSELL SEH, HICKEY GI, LOWRY WS, WHITE P AND ATKINSON RJ. (1990). Allele loss from chromosome 17 in ovarian cancer. *Oncogene*, **5**, 1581–1583.

SAITO H, INAZAWA J, SAITO S, KASUMI F, KOI S, SAGAE S, KUDO R, SAITO J, NODA K AND NAKAMURA Y. (1993). Detailed deletion mapping of chromosome 17q in ovarian and breast cancers: 2-cM region on 17q21.3 often and commonly deleted in tumors. *Cancer Res.*, **53**, 3382–3385.

SAITO S, SAITO H, KOI S, SAGAE S, KUDO R, SAITO J, NODA K AND NAKAMURA Y. (1992). Fine-scale deletion mapping of the distal long arm of chromosome 6 in 70 human ovarian cancers. *Cancer Res.*, **52**, 5815–5817.

SLAMON DJ, GODOLPHIN W, JONES LA, HOLT J, WONG SG, KEITH DK, LEVIN WJ, STUART SG, UDOVE J, ULLRICH A AND PRESS MF. (1989). Studies of the HER-2/*neu* proto-oncogene in human breast and ovarian cancer. *Science*, **244**, 707–712.

STACK M, JONES D, WHITE G, LISCIA DS, VENESIO T, CASEY G, CRICHTON D, VARLEY J, MITCHELL E, HEIGHWAY J AND SANTIBANEZ-KOREF M. (1995). Detailed mapping and loss of heterozygosity analysis suggests a suppressor locus involved in sporadic breast cancer within a distal region of chromosome band 17p13.3. *Hum. Mol. Genet.*, **4**, 2047–2055.

TAVASSOLI M, RUHRBERG C, BEAUMONT V, REYNOLDS K, KIRKHAM N, COLLINS WP AND FARZANEH F. (1993). Whole chromosome 17 loss in ovarian cancer. *Genes Chrom. Cancer*, **8**, 195–198.

TSAO SW, MOK CH, OIKE K, MUTO M, GOODMAN HM, SHEETS EE, BERKOWITZ RS, KNAPP RC AND LAU CC. (1991). Involvement of p53 gene in the allelic deletion of chromosome 17p in human ovarian tumors. *Anticancer Res.*, **11**, 1975–1982.

WALES MM, BIEL MA, ELDEIRY W, NELKIN BD, ISSA P, CAVENEE WK, KUERBITZ SJ AND BAYLIN SB. (1995). p53 activates expression of HIC-1, a new candidate tumour suppressor gene on 17p13.3. *Nature Med.*, **1**, 570–577.

WAN M, ZWEIZIG S, D'ABLAING G, ZHENG J, VELICESCU M AND DUBEAU L. (1994). Three distinct regions of chromosome 6 are targets of loss of heterozygosity in human ovarian carcinomas. *Int. J. Oncol.*, **5**, 1043–1048.

YANG-FENG TL, HAN H, CHEN K-C, LI S, CLAUS EB, CARCANGIU ML, CHAMBERS SK, CHAMBERS JT AND SCHWARTZ PE. (1993). Allelic loss in ovarian cancer. *Int. J. Cancer*, **54**, 546–551.